# A Novel Telomerase Activator Suppresses Lung Damage in a Murine Model of Idiopathic Pulmonary Fibrosis

**DOI:** 10.1371/journal.pone.0058423

**Published:** 2013-03-14

**Authors:** Claude Jourdan Le Saux, Philip Davy, Christopher Brampton, Seema S. Ahuja, Steven Fauce, Pooja Shivshankar, Hieu Nguyen, Mahesh Ramaseshan, Robert Tressler, Zhu Pirot, Calvin B. Harley, Richard Allsopp

**Affiliations:** 1 University of Texas Health Science Center at San Antonio, San Antonio, Texas, United States of America; 2 John A. Burns School of Medicine, University of Hawaii, Honolulu, Hawaii, United States of America; 3 Geron Corporation, Menlo Park, California, United States of America; 4 Cellerant, Redwood City, California, United States of America; 5 Beckman Coulter, Inc., Brea, California, United States of America; McMaster University, Canada

## Abstract

The emergence of diseases associated with telomere dysfunction, including AIDS, aplastic anemia and pulmonary fibrosis, has bolstered interest in telomerase activators. We report identification of a new small molecule activator, GRN510, with activity *ex vivo* and *in vivo*. Using a novel mouse model, we tested the potential of GRN510 to limit fibrosis induced by bleomycin in *mTERT* heterozygous mice. Treatment with GRN510 at 10 mg/kg/day activated telomerase 2–4 fold both in hematopoietic progenitors *ex vivo* and in bone marrow and lung tissue *in vivo,* respectively. Telomerase activation was countered by co-treatment with Imetelstat (GRN163L), a potent telomerase inhibitor. In this model of bleomycin-induced fibrosis, treatment with GRN510 suppressed the development of fibrosis and accumulation of senescent cells in the lung via a mechanism dependent upon telomerase activation. Treatment of small airway epithelial cells (SAEC) or lung fibroblasts *ex vivo* with GRN510 revealed telomerase activating and replicative lifespan promoting effects only in the SAEC, suggesting that the mechanism accounting for the protective effects of GRN510 against induced lung fibrosis involves specific types of lung cells. Together, these results support the use of small molecule activators of telomerase in therapies to treat idiopathic pulmonary fibrosis.

## Introduction

Telomeres are genetic elements that cap and protect the ends of eukaryotic chromosomes. The enzyme telomerase is a specialized reverse transcriptase that functions to extend telomeres in proliferating cells. Most somatic cells in adult humans lack sufficient levels of telomerase, and consequently, telomere reserve, the total amount of all telomeric DNA in the genome, is gradually depleted during aging [1]. Some cell types, such as hematopoietic cells [2] and certain types of stem cells [3], [4], express telomerase, which can slow the rate of telomere attrition. If left unabated, the depletion of telomeric reserve will eventually cause the critical shortening of 1 or more telomeres, and cause cell senescence [1], [5]. Re-activation of telomerase in human cells, for example by genetic manipulation of the expression of telomerase reverse transcriptase (TERT), the catalytic component of telomerase, can sufficiently enhance telomerase activity to prevent the loss of, or even increase, telomeric reserve in cells and prevent cell senescence [6]. Within the past decade, a number of diseases or conditions have been associated with mutations in telomerase and/or accelerated telomere loss, including AIDS [7], dyskeratosis congenita [8], [9], aplastic anemia [10] and idiopathic pulmonary fibrosis (IPF) [11], [12], [13].

IPF is a chronic and often fatal disorder with unknown etiology. It affects over 5 million people worldwide with 40,000 deaths per year (the same as breast cancer). Due to limited therapeutic success the probability of a 5-year survival is approximately 50%. The disease is typically diagnosed in middle-aged patients. IPF is described as an aberrant wound healing response after recurrent alveolar injury [14], [15], where restitution of the epithelial integrity and the restoration of tissue structure and function, important steps in normal healing, are compromised. Proliferation and repopulation of denuded alveolar epithelial cells (AEC) is not observed, while excessive AEC apoptosis has been reported [16]. Moreover, shorter telomere length that over time leaves the epithelium susceptible to senescence has also been reported in sporadic IPF patients. In addition, some patients have a familial disorder that segregates as a dominant trait with incomplete penetrance [17], [18]. Among the disease-causing genes in familial IPF, mutations in the genes encoding telomerase (Tert and the RNA component of telomerase (TR)) are found in approximately 15% of cases [19], [20]. Both Tert and TR are required to maintain telomere integrity. In affected individuals, telomerase activity is reduced about 50% and accelerated telomere loss is seen. Mutations in Tert or TR that result in telomere shortening over time confer a dramatic increase in susceptibility to adult-onset IPF [12].

Recently, a small molecule activator of telomerase, cycloastragenol (GRN665 or TAT2; Figure S1), has been identified and shown to enhance telomerase activity and increase telomeric reserve in human lymphocytes in vitro [21]. In the present study, we have assessed the therapeutic potential of GRN510, a new activator of telomerase, to protect against the development of pulmonary fibrosis using a murine model of induced pulmonary fibrosis.

## Materials and Methods

The mice were fed with a standard diet and maintained in a temperature and light-controlled room (22 uC,14L:10D; light starting at 0700 h), in accordance with the guidelines of the Laboratory Animal Services at the University of Hawaii and the Committee on Care and Use of Laboratory Animals of the Institute of Laboratory Resources National Research Council (DHEW publication 80–23, revised in 1985). The University of Hawaii at Manoa IACUC committee has specifically approved this study.

### Animals and Induction of Fibrosis

**Figure 1:**
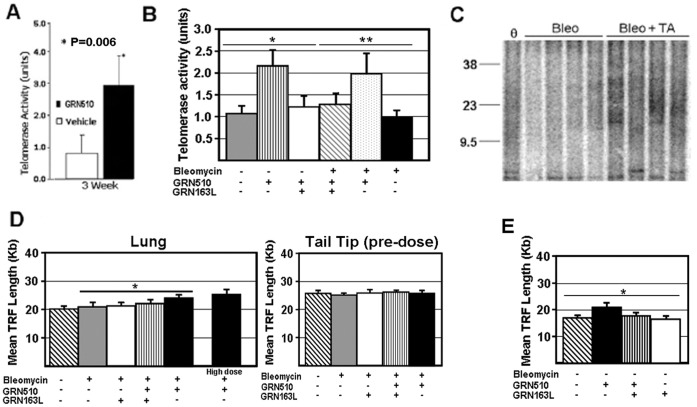
Treatment with GRN510 enhances telomerase function in the lung using a bleomycin-induced model of pulmonary fibrosis. A. Analysis of telomerase activity levels in lung tissue for Tert Het mice dosed with GRN510 at 10 mg/kg/d for 21 days post-Bleomycin treatment (23 days total). Intratracheal injection of Bleomycin was performed on day 0. The mean telomerase activity level for the GRN510 treated and vehicle control mice are shown (n = 4–10 per group). The average activity level for the vehicle control group was arbitrarily assigned a value of 1.0. Asterisk, * denotes significant difference relative to the vehicle control mice (Student's t Test). **B.** Analysis of the effect of telomerase inhibitor GRN163L on GRN510-mediated telomerase activation in the lung. Mice were dosed with GRN510 (10 mg/kg/d) and/or the telomerase inhibitor GRN163L (13 mg/kg/d) as indicated for 21 days (n = 5–10 for all groups). The average activity level for the vehicle control group was arbitrarily assigned a value of 1.0. Asterisks, (*, **) denotes significance amongst the indicated groups (pvalue≤0.03, 1-way ANOVA; P≤0.01 comparing GRN510+GRN163L treated group to GRN510 treated group Student’s t Test). **C.** Sample southern blot for analysis of terminal restriction fragment length of lung tissue after GRN510 (TA-telomerase activator) treatment. A control sample for untreated Tert Het mice is represented by 'θ'. Size of molecular weight standards are shown on the left (Kb). **D.** Quantitative analysis of mean TRF length lung tissue after 3 weeks of GRN510 treatment. For each group, n = 5–10. Asterisk * denotes significance amongst indicated groups; P = 0.03, 1-way ANOVA). **E.** Mean TRF length analysis of PBS control mice. Analysis was performed after 3 weeks of dosing (all mice dosed as described above). N = 4 per group. Asterisk, * denotes significance amongst groups (P = 0.02, 1-way ANOVA). For all plots, mean ± SEM is shown.

Animal experiments were conducted using a protocol approved by the Institutional Animal Care and Use Committee of the University of Hawaii. Heterozygote TERT mouse colony (Tert Het ) [22] was maintained at the University of Hawaii. The colony was maintained by Het X Het crossings, and was at 13–14 generation of crosses at the time of this study. Mean TRF length has been observed to be within the ∼17–21 Kb size range across multiple generations. Analysis of telomere length, prior to treatment, in the animals used for all experiments showed equivalent average telomere lengths for all groups (Figure 1).

Pulmonary fibrosis was induced with bleomycin by surgically exposing the tracheae of 8–12 week old mice and confirming direct tracheal instillation. Mice were dosed by subcutaneous injection with PBS or 1.3 U/kg body weight of bleomycin sulfate (Calbiochem, EMD Chemicals, US) dissolved in PBS. Progression of fibrosis was monitored by sacrificing control and treated mice at days 7, 14 and 21 following bleomycin injury.

Euthanasia was performed with gradual filling of a chamber with CO_2_. Bleeding from the nose, gross or histological evidence of CO_2_-induced pulmonary hemorrhage was not observed under these conditions.

### Measurement of Lung Mechanics

Dynamic compliance was measured using a Flexivent instrument (Scireq Inc., Canada; http://www.scireq.com/products/flexivent) as previously described [23], [24]. Briefly, mice were weighed prior to anesthesia with ketamine (100 mg/kg i.p.) and xylazine (10 mg/kg i.p.). Tracheae were exposed surgically, cannulated, and attached to the Flexivent for ventilation. The positive end expiratory pressure (PEEP) was set at 2 cm H_2_O. The mice were mechanically ventilated at 150 breaths/min and a tidal volume of 7.5 ml/kg. Resistance, compliance, and elastance were measured 15 times over 45 sec after an intra-peritoneal (i.p.) injection of pancuronium bromide (Sigma, St. Louis, MO, USA). The data were analyzed using the Scireq single compartment model. The measurements taken were categorized as forced maneuvers. After measurements, mice were disconnected from the ventilator, sacrificed by CO_2_ inhalation and exsanguinated by cardiac puncture. Bronchoalveolar lavage (BAL) was performed with 1 ml of PBS. The left lung was then excised for paraffin sectioning, whereas the right lung was reserved for frozen sectioning and RNA and protein extraction. The particular lobe selection for each assessment remained consistent throughout the experiment.

### Analysis of Telomerase Activity

Telomerase activity was quantitatively assessed by the telomere repeat amplification protocol (TRAP) assay using the TRAPeze telomerase detection kit (Millipore) as described [3]. For each TRAP reaction, either 1000 cell equivalents (bone marrow extracts) or 1 ug of protein (lung extracts) was used.

### Telomere Length Analysis

Telomere length was measured by Southern analysis of terminal restriction fragment (TRF) length using field inversion gel electrophoresis (FIGE) as described previously [25].

### Bone Marrow Isolation, Immunostaining & Hematopoietic Progenitor FACS Sorting

The procedure for preparation of bone marrow for cytometric analysis and sorting of hematopoietic progenitor cells is as described previously [25].

### Histology, Collagen Staining, and Immunohistochemistry

Non-inflated lung tissues were fixed in a solution of PBS containing 4% paraformaldehyde. Lungs were embedded in paraffin. Five-micron thick paraffin sections, layered on silane-coated slides, were used for hematoxylin and eosin staining and examine the pathological changes. To quantify collagen deposition, lung sections were stained with picrosirius red (PSR). To minimize variation, samples were processed under identical conditions in as minimal number of batches as practical. To quantify collagen networks in lung parenchyma of TERT^+/−^ mice, the level of picrosirius red staining was assessed with ImagePro software v6.2 (Media Cybernetics,Inc. Bethesda, MD, USA) and expressed as a percentage of the total area of the image analyzed as previously described [26].

Immunohistochemical staining for α-smooth muscle actin (αSMA) and the senescence marker MH2A was carried out using paraffin embedded tissue sections. Briefly, deparaffinized and rehydrated sections were incubated in boiling water bath in 10 mM citrate buffer for 20 minutes for antigen retrieval. Peroxidase activity was quenched by incubation of 0.3% H_2_O_2_ in methanol for 30 min. After blocking with Vectastain Normal Serum using Vectastain ABC Kit (Vector Laboratories, Inc. Burlingame, CA USA) sections were incubated overnight under moist conditions at 4°C with assorted primary antibodies diluted in Vectastain normal Serum (Vector Laboratories, Inc.). Sections were then incubated at room temperature for 1hour with Vectastain Biotinylated secondary antibody (Vector Laboratories Cat. No.: PK 4001). After a PBS wash, slides were then incubated in Vectastain ABC reagent (Vector Laboratories) for 30 minutes, and then incubated with HistoMark BLACK (KPL, Inc. Gaithersburg, MD USA) substrate solution, reconstituted in enhance blank buffer, for 10 minutes. Sections were counterstained with hematoxylin, dehydrated and mounted.

### Collagen Deposition Quantification

For evaluation of collagen content, we used the Sircol Collagen Assay kit (Biodye Science, Westbury, NY) according to the manufacturer's instructions. Briefly, 10 mg of whole lung tissue was homogenized in 500 ml of extraction buffer™ and incubated for 12 hr at 4°C with stirring. Tissue homogenates were spun at 15,000×g for 60 min at 4°C. Aliquots of lung homogenate were then assayed for total collagen levels by comparison with a standard curve of collagen obtained by optical density at 540 nm with a SpectraMAX 340 (Molecular Devices, Toronto, Canada).

### FACS Analysis of Fibrocytes

Digestion of the lungs was performed in a similar way as previously described [27]. Bone Marrow (BM) cell suspension was obtained from femurs from mice. Cells obtained from blood, BM and lungs were resuspended in FACS buffer, pelleted and incubated with murine Fc Blocking (BD Biosciences) before staining. Cells were initially stained with CD184 (PE), and CD45 (FITC) (BD Biosciences). After surface staining, cells were permeabilized with cytofix/cytoperm (BD Biosciences), stained with a goat primary antibody against collagen I (SantaCruz, Santa Cruz, CA), followed by detection with APC-conjugated donkey anti goat IgG. Acquisition and analysis of the data was done on a FACSCalibur using CELLquest Pro.

### Gene and Protein Expression

Total RNA was extracted from cell or tissue samples using the RNeasyTM kit (Qiagen Inc., Valencia, VA, USA). RNA samples were treated with 0.05 U/ml of DNase I (Qiagen) at 20°C for 15 min. Total RNA (5 µg) was converted into first strand cDNA using random hexamers (SuperScript First-Strand Synthesis System for RT-PCRTM, Invitrogen Life Technologies, Carlsbad, CA). The level of expression of alpha 2 chain of type I procollagen (*procol1A2)*, *SIRT6*, and *p21* mRNA was detected by quantitative RT-PCR (qPCR) using commercially available TaqMan probes (Applied Biosystems, Foster City, CA). Total protein was extracted by homogenizing one whole lobe of frozen lung tissues on ice in 10 ml of Cell Lytic MT buffer (Sigma, St. Louis, MO) containing 1 mM DTT, 1X protease inhibitor cocktail (Calbiochem, San Diego, CA), and 5 mM EDTA. Homogenates were centrifuged at 16,000 g and the protein concentration in the supernatant was determined by the Bradford assay (Bradford Reagent, Bio-Rad). Total protein (25 µg) was combined with reducing Laemmli buffer, heated at 95°C for 5 min, cooled on ice, and loaded into wells of a 10% polyacrylamide gel (Bio-Rad). Protein was transferred to PVDF membrane and blotted with primary antibody including anti- p53 and anti α-smooth muscle actin. Appropriate ECL-peroxidase-linked secondary antibodies were detected using ECL Plus (Amersham, Piscataway, NJ). For densitometry, digital images of autoradiographic film were captured using a flat bed scanner (CanoScan 8400 F, Canon USA Inc.). The net intensity of the target bands was normalized to that of the β-actin band to obtain a relative level of proteins of interest on an unsaturated exposure. For expression analysis of cellular senescence markers, p16^INK4a^ (p16), p21^WAF1/CIP1^ (p21), cells treated in culture were lysed in M-Per lysis buffer (Pierce, cat. # 78503) plus 1 mM of PMSF and RNasin (1 u/uL), and the lysates were spun for 20 min using bench top centrifuge at 14000 rpm, 4C. Five uL of the extract were used for P16 & p21 expression analysis using AgPath-ID One-Step RT-PCR kit (ABI Cat #AM1005) with commercially available TaqMan gene expression primer & probe sets from ABI. Total RNA concentration of the cell extract were measured by RiboGreen® RNA Quantitation Kit (molecular Probes, Cat. R-11490) and used for gene expression normalization.

### Senescence–associated β*-*galactosidase Analysis

Senescence was evaluated by SA-βgal activity as described by Debacq-Chainiaux, et al [28]. Briefly, lungs were flash frozen in liquid nitrogen and immediately sectioned at 10 µm. Tissue was fixed with 4% paraformaldehyde with 30% sucrose for 5 minutes and then washed. Slides were incubated over night at 37°C in staining solution (40 mM citric/acid/sodium phophate buffer, 5 mM potassium hexacyano-ferrate (II) trihydrate, 5 mM potassium hexacyano-ferrate (III), 150 mM sodium chloride, 2 mM magnesium chloride, 1 mg/ml X-gal; pH 6.0), then washed in PBS. Senescent cells stained blue at a pH 6.0 and was visualized by light microscopy.

### *In vitro* Assessment of Telomerase Activation with GRN510

Human small airway epithelial cell (SAEC) were obtained from ScienCell Research Laboratories and human lung fibroblast cell, IMR-90, was purchased from ATCC [29], [30]. Cells were cultured in the commercial recommended condition. SAEC at population doubling 5–14 were used for the in vitro assessment of telomerase activation by GRN510. Cells were seeded in 24-well plates and treated with various amounts of GRN510 in 0.1% of DMSO for 48–72 hrs. Cells were lysed and extracts prepared in CHAPS buffer (50–100 uL) per manufacturers’ protocol (TRAPeze kit; Millipore).

### Statistical Analysis

One-way ANOVA was used to determine statistical significance amongst groups and Student’s t Test was used in some instances for comparisons between specific groups. Data entry, management, and statistical analysis were performed using Prism software (GraphPad Software, San Diego, CA, USA).

## Results

### Small Molecule Telomerase Activators

Cycloastragenol (GRN665 or TAT2) a small molecule isolated from the roots of the plant *Astragalus membranaceus* (Fig. S1), and activates telomerase in multiple types of human cells in culture including human lymphocytes [21]. GRN510 is a novel and proprietary chemical entity derived from GRN665/TAT2, and is available from Geron Corporation under a Materials Transfer Agreement.

### GRN510 Activates Telomerase in *In Vivo*

We initially assessed the ability of GRN510 to activate telomerase *in vivo* by measurement of telomerase activity in bone marrow (BM) progenitor cells after a daily regimen of treatment of mice with GRN510 for 1 week. This and all subsequent experiments, except where indicated, were performed in mice heterozygous for the *Tert* gene (Tert Het mice) [22], a strain which has attenuated levels of telomerase activity analogous to individuals with defects in Tert[8]–[9]. Compared to control mice receiving vehicle alone or untreated mice, telomerase activity levels were significantly increased in BM progenitor cells at both high (20 mg/kg/d) and low (10 mg/kg/d) dose (Fig. S2A; P≤0.04, 1-way ANOVA). Comparison of BM progenitor cell numbers for the GRN510 treated mice and vehicle control mice revealed no significant difference, showing that the GRN510-induced levels of telomerase activity was likely due to effects on telomerase as opposed to changes in proliferation or cell phenotype (Fig. S2B).

To assess the potential of GRN510 to activate telomerase in lung tissue, we treated Tert Het mice with GRN510 (10 mg/kg/day) for 23 days (day -2 through day 21), conducted a single intratracheal injection of bleomycin on day 0. In agreement with the elevated level of telomerase in the BM progenitor cells of GRN510 treated mice (10 mg/kg/day), we observed a significant and sustained enhancement of telomerase activity in the lung tissue of the GRN510 treated mice (Fig. 1A; P = 0.006). Enhanced telomerase activity levels were observed in both bleomycin treated and PBS control mice following dosing with GRN510, which was repressed upon co-treatment with the potent telomerase inhibitor GRN163L (13 mg/kg/d) (Fig. 1B). To determine whether the level of telomerase activation we observed has a physiologically relevant effect on telomeres, we assessed telomere length in lung tissue by Southern analysis of terminal restriction fragment (TRF) length. Telomeres in the GRN510 treated mice (10 mg/kg/d) were ∼15% (∼3.0 Kb; P = 0.03, 1-way ANOVA) longer relative to vehicle control mice, untreated Tert Het mice, or mice treated with GRN163L or with both GRN510 & GRN163L (Fig. 1D), indicating that GRN510 mediated induction of telomerase activity is sufficient to repair telomeres in vivo. In addition, mice receiving a high dose of GRN510 (20 mg/kg/d) also exhibited telomere length extension relative to vehicle control or untreated mice (Fig. 1D; P = 0.02, 1-way ANOVA). This was not attributed to happenstance differences in initial telomere lengths prior to treatment amongst the different groups of mice, since telomere length of tail tip DNA prior to dosing showed approximately equal average telomere lengths for all groups. Analysis of total telomeric signal intensity, another measure of total telomere length, on the Southern blots also revealed significantly higher (1-way ANOVA; P = 0.01; not shown) average telomere length in the GRN510 treated group relative to all other control groups. Significant extension of telomere length was also observed in the GRN510 treated group in PBS control mice as well (Fig. 1E; 1-way ANOVA; P = 0.02). Together these data support a telomerase dependent effect of GRN510 on telomere length in Tert Het mice following bleomycin induced fibrosis.

### GRN510 Mediates Attenuation of Induced Pulmonary Fibrosis in Telomerase-Compromised Mice in a Dose Dependent Manner

**Figure 2:**
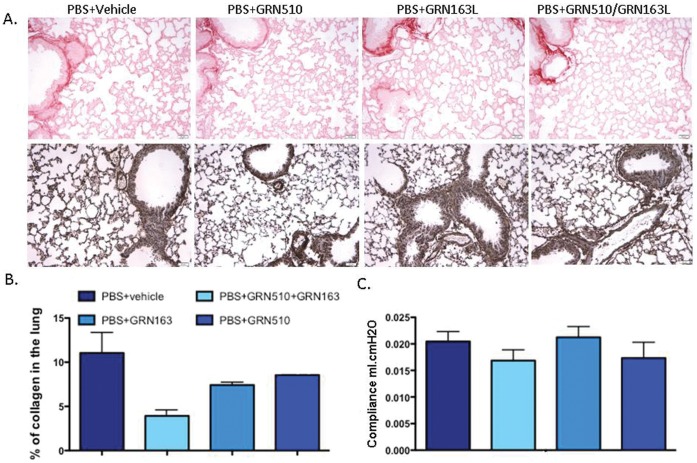
GRN510 and GRN163L treatments did not affect the structure and the function of the lungs. Mice were treated with GRN510, GRN163L, and GRN510+ GRN163L for 3 weeks. Expression of α-smooth muscle actin (αSMA) and collagen deposition was evaluated as well as the baseline lung mechanics. **A.** Representative picorsirius red staining (upper panel) and immunostaining of α−SMA (lower panel) are shown; X200. **B.** Collagen deposition was quantified using ImagePro software by blinded personnel to the treatment groups. **C.** Dynamic compliance (ml/cm H2O) was measured. Treatment with the 2 components, alone or in combination indicated no difference in compliance among groups. Statistical analysis was performed by 1-way ANOVA (Prism, GraphPad Software, Inc). N = 4 per group (same animals as assessed in Fig. 1E); For all plots, mean ± SEM is shown.

To assess the effect of telomerase activation of pulmonary fibrosis (PF), we performed an intratracheal injection of bleomycin (day 0) to our PF model in telomerase-compromised mice (Tert Het strain), and subsequently assessed the potential of a daily dosing regimen of GRN510 at 10 mg/kg/d or 20 mg/kg/d, beginning at day -2, to attenuate the development of the fibrotic phenotype in the lungs. Initial histological and functional evaluation of the effects of GRN510 or the potent telomerase inhibitor GRN163L (13 mg/kg/d), either alone or together, in the lung tissue of PBS-treated control mice (no bleomycin injury) showed no appreciable effects (Fig. 2).

**Figure 3:**
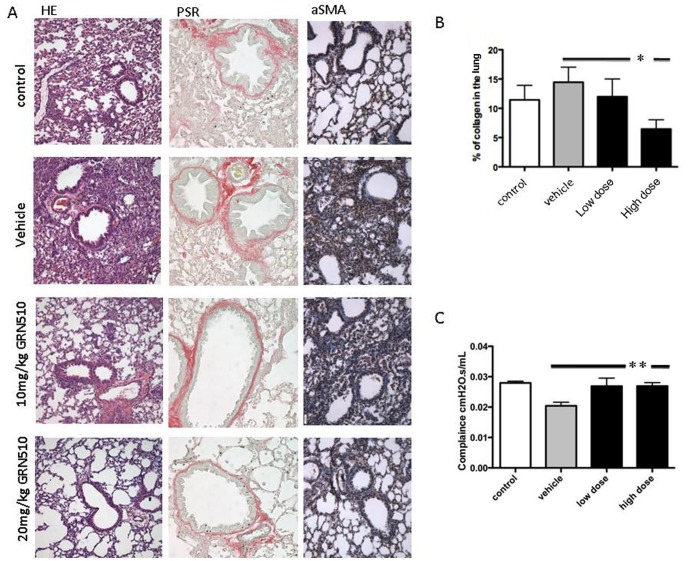
Reduced fibrotic response in bleomycin-injured lungs after treatment with GRN510. A. Tert Het mice were treated with GRN510 for 3 weeks at the indicated dose and subjected to bleomycin-induced fibrosis (day 0). Representative histological sections from the whole left pulmonary lobe were taken from each animal after 3 weeks of dosing and stained with H&E (Left panel), picrosirius red (middle panel), and a-SMA (Right panel). All these stainings indicate that treatment with GRN510 attenuates the bleomycin injury and in a dose dependent manner. **B.** Levels of picrosirius red staining were quantified with ImagePro software. Asterisk, * denotes significance amongst indicated groups (P = 0.01, 1-way ANOVA) **C.** Dynamic compliance (ml/cm H2O) was significantly increased in the lungs of bleomycin-treated Tert Het mice. This increase in compliance was attenuated after GRN510 treatment 3 weeks after bleomycin injury (P = 0.01; 1-way ANOVA). N = 4–10 animals per group, values given are mean ± SEM.

In mice treated with GRN510 following bleomycin induced injury to the lung, histological evaluation of H&E stained lung tissue sections 3-weeks post-bleomycin injury indicated a marked reduction in inflammatory infiltrate and remodeling after GRN510 treatment, which was noticeably more pronounced in the 20 mg/kg treatment group than in the 10 mg/kg treatment group (Fig. 3A). The hallmark of pulmonary fibrosis is the accumulation of collagen in the parenchyma. We quantified collagen deposition after picrosirius red staining. The increase of collagen deposition observed after bleomycin injury was not observed in mice treated with 20 mg/kg/d GRN510 (Fig. 3A,B; P = 0.01; 1-way ANOVA), and corresponded with longer telomeres in the GRN510 treated mice relative to the vehicle or GRN510+ GRN163L controls (Fig. 1). We also measured collagen deposition by analysis of α-smooth muscle deposition, which showed noticeably decreased levels present in the lungs of mice treated with 20 mg/kg/d of GRN510 (Fig. 3A), in agreement with the H&E staining and picrosirius red analysis of collagen. In addition, analysis of lung collagen levels by Sircol analysis also showed reduced collagen accumulation in mice treated with GRN510 following bleomycin injury as compared to vehicle control or mice co-treated with GRN163L (Table 1). Analysis of lung compliance, using the Flexivent™ instrument (Scireq), in these same mice immediately prior to these end-point analyses (3 weeks post-bleomycin injury) also showed significant improvement of lung function in mice treated with GRN510 (Fig. 3C; P = 0.01, 1-way ANOVA).

**Table 1:** Evaluation of collagen content in lung tissues (Ug of collagen/mg of tissue).

Treatment		Het Tert	WT
PBS	Vehicle	27.36±5.7	25.77±1.57
	GRN510*	25±2.3	25.96±1.19
	GRN163L	26.49±1.72	25.85±0.69
	GRN510+GRN163L*	31.66±2.18	27.89±2.05
Bleomycin	Vehicle	38.69±1.02	
	GRN510*	31.4±3.3	
	GRN163L	34.25±10.44	
	GRN510+GRN163L*	42.66±1.92	

* All mice were dosed at 10 mg/kg/day of GRN510.

**Figure 4:**
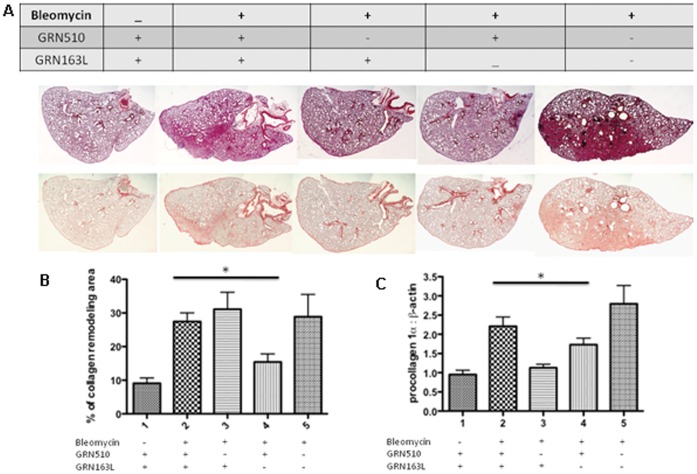
The GRN510-mediated protection from bleomycin-induced fibrosis is dependent on telomerase activation. Tert Het mice subjected to Bleomycin-induced fibrosis were treated with GRN510 (10 mg/kg/d) and the telomerase inhibitor GRN163L as indicated. **A.** Representative histological sections were obtained from the whole left pulmonary lobe taken from each animal 11 days after bleomycin-induced injury and stained with H&E (Top panel) or with picrosirius red (Lower panel). To minimize variation, samples were processed under identical conditions. **B.** Levels of picrosirius red staining were quantified with ImagePro software. Asterisk, * indicates significance amongst indicated groups (P = 0.02, 1-way ANOVA). **C.** RT-PCR expression levels of procollagen1α mRNA in the lung. Analysis was performed in triplicate for each specimen. Asterisk, * denotes significance amongst indicated groups (P = 0.04, 1-way ANOVA). N = 5–10 animals per group; for all plots, mean ± SEM is shown.

To verify that The GRN510 mediated repression of induced fibrosis was a direct affect of telomerase activation, we repeated the bleomycin induction of fibrosis in Tert Het as described above and included additional groups of mice treated with the telomerase inhibitor GRN163L or both GRN510+ GRN163L (10 mg/kg/d and 13 mg/kg/d respectively). Significant repression of collagen deposition was only observed in the GRN510 treated group (1-way ANOVA P = 0.01) and not observed in the GRN510+GRN163L treated group (P = 0.04; 1-way ANOVA) (Fig. 4). These data suggest that the ability of GRN510 to suppress induced pulmonary fibrosis is dependent on its ability to activate telomerase.

### GRN510 Affects Collagen Deposition in bleomycin-Injured Wild Type Mice

As additional confirmation of the potential relevance of these results with Tert Het mice to patients with PF, we have assessed the ability of GRN510 on preventing fibrotic injury in the lungs using wild type (WT) mice. In this experiment, we also detected a significantly attenuated collagen deposition in the lung tissue from the GRN510 treated group (8.9±3.1%) relative to the bleomycin vehicle control group (21.95±3.6%; P = 0.02 Student’s t Test; Fig. S3A). Similar to Tert Het mice, PBS saline controls did not exhibit any noticeable effect of GRN510 overall (Fig. S3B–D and data not shown). Together, these observations all indicate that telomerase activation may be effective at alleviating hallmarks of PF.

### The Inflammatory Response to Bleomycin is not Affected by GRN510 Treatment

As an initial step to assess the mechanism of GRN510 protective effect, we measured immune cell infiltration into the lungs via analysis of total cell counts from BAL fluid following bleomycin challenge. In both *Tert Het* and *WT* animals treated with GRN510 (10 mg/kg/d), the inflammation response appeared within similar range (Fig. S4A and B). Profile analysis of immune cells infiltrating into the lungs post-bleomycin injury revealed that all the groups responded with increased infiltration of neutrophils and lymphocytes into the lungs. The proportion of the infiltrating cells was not altered by the drug treatment (Fig. S4C). In addition, analysis of circulating fibrocytes in the lung and blood revealed no affect in the GRN510-treated group as compared to vehicle control mice (Fig. S4D). Therefore, the difference in the fibrotic response could not be explained by a reduced or lack of inflammatory response.

### GRN510 Protects Against Senescence in Alveolar Epithelial Cells

**Figure 5:**
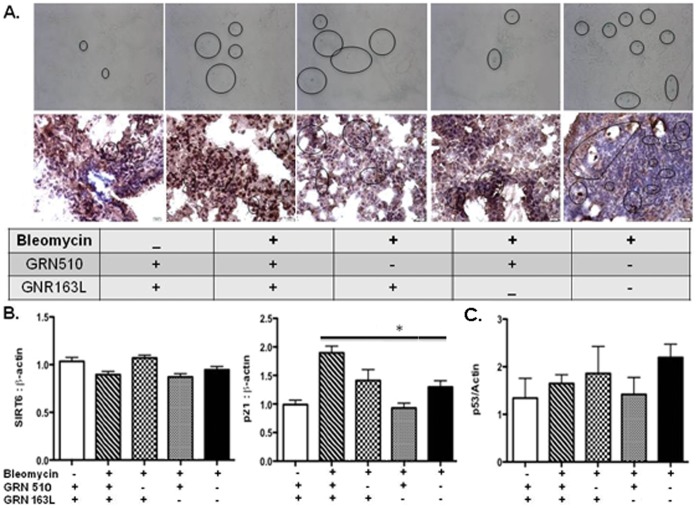
Reduced cellular senescence in bleomycin-injured lung tissue with GRN510 treatment. All analyses were performed on the same mice as in Fig. 4. **A.** Analysis of SA-β galactosidase activity in the lung tissue. Shown are representative paraffin-embedded lung sections after staining for SA-β gal activity (blue staining). Increased SA-β gal activity was detected in the mice challenged with bleomycin and treated with vehicle or GRN510 and GRN163L. In contrast, reduced SA-β gal activity was detected in the mice challenged with bleomycin and treated with GRN510. Positive stained cells are encircled. Magnification 40X. Representative paraffin embedded lung sections immunohistochemically stained for MH2A using enhance blank staining solution (KPL, Gaithersburg, MD); n = 3 different specimens with 3 fields of view analyzed per section; Hematoxylin was used as a negative counter-stain. Magnification of 40X. Circles denote positive staining. **B.** Analysis of *SIRT6* and *p21* expression in the lung. Targeted genes and β-actin expression at the mRNA level was determined by qRT-PCR. Analysis was performed in triplicate for each specimen. N = 5–10 animals per group. Asterisk, * indicates significance amongst indicated groups (P = 0.002, 1-way ANOVA). **C.** Western analysis of p53 expression. A trend in increase expression was associated with bleomycin treatment that was partially reverted by GRN510 treatment. For all plots mean ± SEM is shown.

To assess whether GRN510 protects against senescence in bleomycin-injured lungs, we performed analysis of senescence associated-βgal activity, the level of mRNA expression for SIRT6, and p21, and protein expression for p53 and MH2A in lung tissue. Repeat analysis of the effect of GRN510 on bleomycin-induced fibrosis in these animals confirmed the suppressive effect (Fig. 4). Reduced numbers of positive-β gal cells were detectable in GRN510-treated Tert Het mice 11 days after bleomycin injury as compared to vehicle treated animals (Fig. 5A). Co-treatment with telomerase inhibitor GRN163L abrogated this reduction, suggesting that activation of telomerase is also sufficient to protect against senescence. No change in SIRT6 mRNA expression (Fig. 5B) or p53 protein levels (Fig. 5C) was detected between groups, however expression of p21 mRNA in GRN510-treated mice as compared to vehicle-treated or GRN163L control groups (1-way ANOVA p** = **0.002; Fig. 5B) was significantly reduced. In addition, reduced expression of MH2A proteins confirmed that GRN510 treated samples exhibited reduced senescence (Fig. 5A). Analysis of senescence markers in saline controls for all groups showed no significant effect of either GRN510 or GRN163L in the absence of injury (Figure S5). Collectively, these data indicated that alveolar epithelial cells from GRN510-treated lungs are protected against fibrosis development and senescence associated with bleomycin injury, and that this protection is dependent on the activation of telomerase.

### GRN510 increases Telomerase Activity and delays Senescence in Human Small Airway Epithelial Cells (SAEC) but not Lung-Derived Fibroblasts

**Figure 6:**
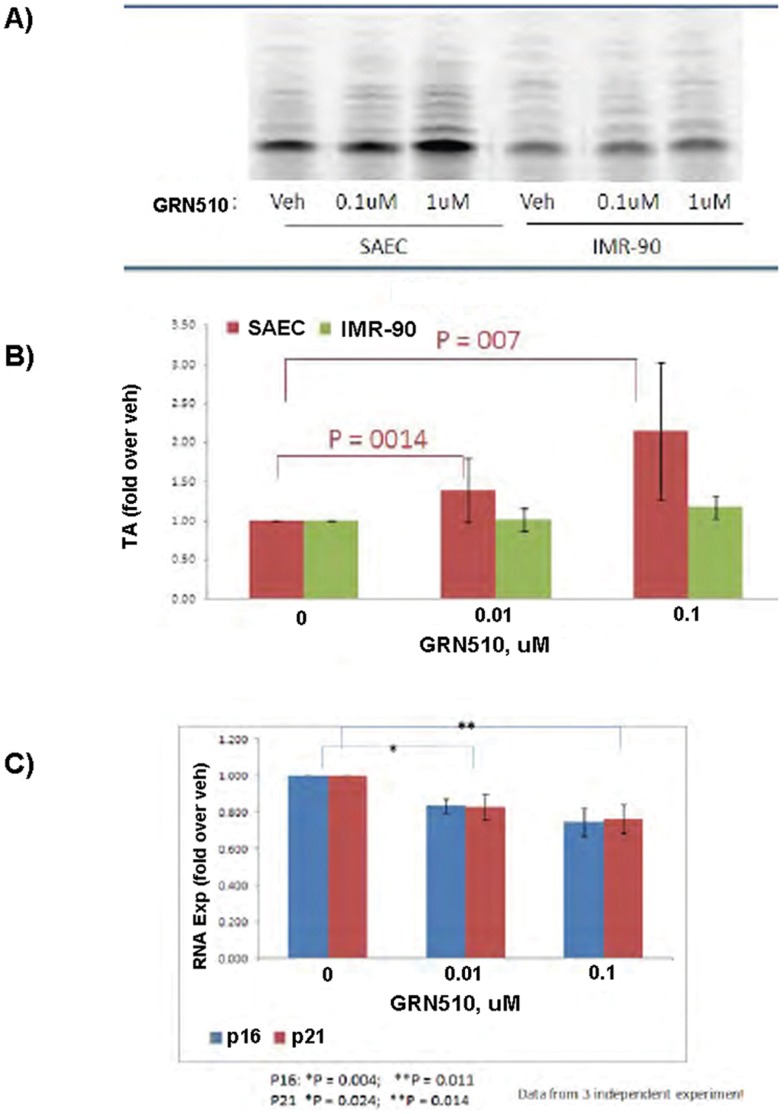
The effect of GRN510 in the lung tissue is cell type dependent. A. Cultured small airway epithelial cells (SAEC) or lung fibroblasts (IMR90) were treated with GRN510 for 48 hours at the indicated doses. Telomerase activity was subsequently assessed in extracts prepared from equal numbers of cells using the TRAP assay. A sample gel analysis of activity is shown. **B.** Quantitation of telomerase activity for the extracts assessed in **A. C.** Analysis of expression of the senescence markers P21 and P16 following long term culture of SAEC cells in the presence of GRN510. Cells were grown continuously in the presence of GRN510 at the indicated doses. Expression was assessed using real time RT-PCR. P values represent Student t Test between indicated groups. For all plots mean ± SEM is shown.

To assess which specific population of lung-derived cells is affected by GRN510, we treated human small airway epithelial cells (SAEC), human fetal lung fibroblast cell line (IMR-90) and primary human lung fibroblasts (hLF) with GRN510 or vehicle for 48 hrs. Telomerase activity was up-regulated 2–4 fold in GRN510 treated SAEC cells, whereas no significant effect was detected in either IMR-90 or hLF (Fig. 6A&B). Both p21 & p16 are reliable markers for cell ageing/senescence. Prolonged culture of SAEC in the presence of GRN510 caused a significant decrease in both p16 and p21 gene expression (P≤0.02)(Fig. 6C). The down regulation of these genes was more prominent in lower passage cells.

## Discussion

We have assessed the potential of a novel small molecule telomerase activator, GRN510, to suppress lung damage in a mouse model of induced fibrosis. Treatment of Tert Het mice with GRN510 induced a sustained enhancement of telomerase levels *in vivo* in both the bone marrow and lung tissue. We have also demonstrated that treatment with GRN510 ameliorated symptoms of induced pulmonary fibrosis in mice, notably, a mitigation of loss of lung function and reduced collagen deposition, without affecting mesenchymal cell activations. In addition our data suggest that the effect of GRN510 involves protection of epithelial cells from undergoing senescence induced by bleomycin.

Treatment of Tert Het mice with GRN510 led to increased telomerase activity in all tissues samples tested (lung and bone marrow; Figure S1 and Figure 1), a modest but significant increase in telomere length, and positive changes in lung structure and function. Treatment of mice with GRN510, or GRN163L, in PBS control mice did not effect lung physiology, collagen expression, or level of senescence markers in Tert Het or WT mice suggesting potential translational outcomes of these drugs in the treatment of pulmonary fibrosis. Since co-treatment of mice with both GRN510 and a potent telomerase inhibitor imetelstat (GRN163L) largely suppressed both the GRN510-mediated activation of telomerase in lung tissue (Fig. 1B) and its protective effect against induced fibrosis (Figure 4), we believe that the principle mechanism of action of GRN510 is mediated through the telomerase and telomere lengthening pathways, as suggested by previous studies [31], [32]. However we cannot exclude the possibility that up-regulation of TERT and its potential role as a transcriptional modulator of the Wnt/β-catenin signaling pathway is also involved [33].

Idiopathic pulmonary fibrosis (IPF) is a chronic and often fatal disorder. Due to limited therapeutic success, the probability of the 3- to 5-year mean survival is approximately 20% in those patients unresponsive to corticosteroids [34], [35], [36]. Over the years, many small molecules have shown promise as potential therapeutic agents to treat IPF based on their ability to inhibit fibrosis in a mouse model, but subsequently failed to in the clinical management studies involving IPF patients, mainly because the drugs were effective to reduce inflammation induced by bleomycin but not in preventing fibrosis [37]. With the bleomycin model, the inflammatory responses driven by bleomycin-induced injury are always an issue due to the non-similarity with human pulmonary fibrosis. However, our data suggest that GRN510 protects the epithelial cells (Fig. 6) from bleomycin injury and although GRN510 reduces the number of infiltrating lymphocytes (Figure S4A), it has little or no effect on inflammatory cell distributions (Fig. S4C). This characteristic makes GRN510 particularly attractive for translation research and applicability for the treatment of IPF patients.

Telomerase was identified as a new target for the treatment of IPF patients in studies showing that mutations in telomerase components, hTERT or hTR, underlie inheritance of IPF in 5–8% of individuals a familial form of IPF [12], [17], [20]. Moreover, telomere length is an indicator of disease onset in the pathogenesis of non-familial forms of IPF [12]. It is crucial to clearly define whether increased telomerase activity affects epithelial injury/repair. Pulmonary fibrosis pathobiology is an aberrant wound healing response to recurrent or stress-induced alveolar injury [38]. The restitution of epithelium integrity is also associated with apoptosis of AEC and fibroblasts. Repeated insults, however, lead to aberrant responses where AEC can no longer proliferate and instead undergo excessive apoptosis and replicative senescence. In contrast, fibroblasts retain proliferative capacity and become resistant to apoptosis, leading to the development of progressive fibrosis [14], [15]. The ability of GRN510 to selectively activate telomerase in SAEC but not in human lung-derived fibroblasts (Fig. 6) suggests a differential response to telomerase activation depending on cell type, therefore potentially allowing a specific drug response on the desired target cells. This observation is supported by the results of others, for example, *TERT null* mice have been shown not to develop fibrosis after bleomycin injury [39], though lung alveolar integrity is compromised in telomerase deficient mice due to increased apoptosis in alveolar epithelial cells type II (AECII) [40]. Other studies suggest that decreased telomerase activity promotes lung fibrosis by enhancing epithelial apoptosis [32]. Together, these data also suggest that the design of truly effective therapies to treat IPF involving telomerase activation warrants careful attention to the specific lung cell type targeted by the drug.

The cells responsible for active deposition of extracellular matrix are the myofibroblasts (fibroblasts characterized by the expression of a-SMA). After bleomycin administration, myofibroblast cell count is increased. Recent data from different labs in regards to the effect of telomerase on myofibroblast differentiation is somewhat conflicting. Some studies have shown that loss of telomerase activity was associated with myofibroblast differentiation [41], [42]. This is in agreement with other results showing over-expression of hTERT in fibroblasts results in potent cell protection against apoptosis [43] and that increased telomerase activity can suppress myofibroblast differentiation [41], [42]. These results are in apparent contrast to that of Liu et al, who found that bleomycin administration in telomerase null mice caused decreased myofibroblast differentiation and no increase in collagen expression [39]. It is possible that myofibroblast differentiation and collagen deposition require telomerase activity, but are triggered by epithelial damage. It is also important to recognize that the origin of myofibroblasts in pulmonary fibrosis is presently unknown, as is the relative role of the newly generated myofibroblasts in pulmonary pathobiology. Thus it remains possible that telomerase deficiency effects myofibroblast differentiation by a fundamentally different mechanism than the effect of telomerase activation.

Therapies for PF based on telomerase activation are also attractive since PF is an aged-related disease. We, and, others have shown that in aged lungs, increased senescence is associated with increase susceptibility to pneumonia for example [44]. In IPF patients, telomerase activity is reduced, leading to accelerated telomere shortening [38]. Importantly, telomere shortening has been reported as one putative mechanism to explain the impaired repair/re-epithelialization process due to the extensive denudation of mucosal epithelial layer during the development of idiopathic pulmonary fibrosis [45], [46]. Therefore, the cell senescence associated with development of PF may be at least partially attributed to accelerated telomere shortening, thereby limiting the tissue renewal capacity. Our in vitro and in vivo data indicated that treatment with GRN510 induces an increase in telomere length (Fig. 1) and prevents senescence-induced in epithelial cells (Fig. 5).

While our studies using murine bleomycin-induced pulmonary fibrosis model indicate that increased telomerase activity is protective against fibrosis. However, there is some contradiction in the published results regarding the role of telomerase in pulmonary fibrosis [12], [32], [39]. Even though this is not the main objective of this project, our data have the potential to elucidate some of this quandary. Our results shown that increased telomerase activation in both TERT Het mice and in WT mice protected against PF by preventing senescence in epithelial cells, in concordance with Fridlender et al. study [32]. Furthermore, it also has been shown that shortening of telomere length compromises lung alveolar integrity [40]. Thus our results and those of others, demonstrate that epithelial cells are sensitive to telomerase activation and that increased telomerase activity is important in the maintenance of epithelial integrity and cell survival [32], [40]. However, Liu et al. showed that telomerase deficiency, specifically, reduced TERT expression levels, is associated with reduced Bleomycin-induced fibrosis, as well as reduced lung fibroblast proliferation and myofibroblast differentiation [39]. Interestingly, we have observed that GRN510 treatment did not enhance telomerase activity in lung fibroblasts or protected against senescence, in contrast to the observed effects of GRN510 on lung epithelial cells (Fig. 6). We propose that the overall contraction between those of Liu et al [39] and ours and others [32], [40] may be the result of fundamentally different effects of GRN510 depending on cell type, specifically, the sensitivity of lung fibroblasts to Tert deficiency, and the protective effects of telomerase activation on SAEC in the context of bleomycin induced injury. Furthermore, it also possible that GRN510 activates telomerase primarily via mechanisms that do not involve the up-regulation of TERT, which may also be dependent upon cell type. Further studies to elucidate the exact mechanism of action of GRN510 in lung epithelial cells and lung fibroblasts will be required to confirm this.

In conclusion, our data show that GRN510 can reduce fibrosis following bleomycin administration in animal models, suggesting that GRN510 may prove effective as a therapeutic for IPF and possibly other diseases involving age-associated fibrosis. Studies in humans will be needed to confirm clinical utility.

## Supporting Information

Figure S1 **Structure of the small molecule telomerase activator cycloastragenol (GRN510).**
(TIF)

Figure S2 **The small molecule GRN510 activates telomerase in vivo. A.** Tert Het mice were dosed at either 10 or 20 mg/kg/day for 3 weeks, and bone marrow progenitor cells (cKit+LinNegSca1+ sub-population, or KLS) were subsequently FACS sorted for analysis of telomerase activity using the TRAP assay. The mean level of activity ± SEM, is shown (n = 4–10 for per group). The average activity level of the untreated control group was arbitrarily assigned a value of 1.0. Asterisks indicate a significant difference amongst indicated groups (* & ** 1-way ANOVA ≤0.04; Student's t test P≤0.01 for GRN510 treated groups versus vehicle control). **B.** FACS analysis of bone marrow progenitor cell numbers after 3 weeks of treatment for the same mice as assessed in **A.** WBM- whole bone marrow. TA- GRN510.
(TIF)

Figure S3 **Histologic examination and collagen deposition levels in Wild type PBS control mice. A)** Wild type mice were dosed with GRN510 (10 mg/kg/d) for 3 weeks and subjected to bleomycin-induced fibrosis (day 2). Levels of picrosirius red staining were quantified with ImagePro software. * denotes significance between indicated groups (P = 0.007; Student t Test). N = 8–10 animals per group. **B)** Representative photomicrographs of H&E and PSR-stained lung sections of wild type PBS control mice treated along with GRN510 and/or GRN163L showing no changes in gross pathology and collagen deposition. **C)** Freshly synthesized collagen levels detected by Sircoll assay in wild type PBS control mice (same mice as in **B**) show no significant difference. **D)** Relative mRNA levels of collagen expression in Wild type PBS control mice (same mice as in **B**). For all plots, N = 4 animals per group. Mean ± SEM is shown. One-way ANOVA revealed no significant difference in collagen levels amongst the groups.
(TIF)

Figure S4 **Analysis of the effect of GRN510 on levels of infiltrating leucocytes following Bleomycin induced lung injury.** Analysis was performed using Tert Het (**A** and **C**; the same mice used in Figure 4, n = 5−10 mice per group) and wild type mice (**B**; the same mice as used in Suppl Figure **S4A**, n = 8−10 animals per group). **D)** Fibrocyte analysis was performed at 11 days post-bleomycin injury in the indicated tissue (the same mice used in figure 4). All GRN510 treated mice received 10 mg/kg/day. Values given are mean ± SEM; asterisk * denotes significance between indicated groups; P  = 0.02, Student’s t Test.
(TIF)

Figure S5 **Senescence markers levels in** ***Tert−/+*** **PBS control mice. A)** Representative photomicrographs of lung sections, from *Tert−/+* PBS control mice treated with GRN510 (10 mg/kg/d) and/or GRN163L (13 mg/kg/d) for 3 weeks, showing no apparent changes in the MH2A levels. **B)** Relative mRNA levels of *p21* and *sirt6* expressions in *Tert−/+* -PBS control mice treated along with GRN510 and/or GRN163L. One-way ANOVA revealed no significant difference in collagen levels amongst the groups. **C)** Western blot analysis and densitometric quantitations of p53 show no significant difference in the levels of p53 in lung homogenates isolated from mice treated with GRN510 and/or GRN163L. For all plots, mean ± SEM is shown; N = 4 animals per group (same mice as analyzed in Figure 2).
(TIF)

## References

[1] Harley CB (1991) Telomere loss: mitotic clock or genetic time bomb? Mutat Res 256: 271–282. 1722017 10.1016/0921-8734(91)90018-7

[2] Buchkovich KJ, Greider CW (1996) Telomerase regulation during entry into the cell cycle in normal human T cells. Mol Biol Cell 7: 1443–1454. 8885238 10.1091/mbc.7.9.1443 PMC275993

[3] Kim NW, Piatyszek MA, Prowse KR, Harley CB, West MD, et al (1994) Specific association of human telomerase activity with immortal cells and cancer. Science 266: 2011–2015. 7605428 10.1126/science.7605428

[4] Chiu CP, Dragowska W, Kim NW, Vaziri H, Yui J, et al (1996) Differential expression of telomerase activity in hematopoietic progenitors from adult human bone marrow. Stem Cells 14: 239–248. 8991544 10.1002/stem.140239

[5] Hemann MT, Strong MA, Hao LY, Greider CW (2001) The shortest telomere, not average telomere length, is critical for cell viability and chromosome stability. Cell 107: 67–77. 11595186 10.1016/s0092-8674(01)00504-9

[6] Bodnar AG, Ouellette M, Frolkis M, Holt SE, Chiu CP, et al (1998) Extension of life-span by introduction of telomerase into normal human cells. Science 279: 349–352. 9454332 10.1126/science.279.5349.349

[7] Effros RB, Allsopp R, Chiu CP, Hausner MA, Hirji K, et al (1996) Shortened telomeres in the expanded CD28-CD8+ cell subset in HIV disease implicate replicative senescence in HIV pathogenesis. AIDS 10: F17–22. 8828735 10.1097/00002030-199607000-00001

[8] Mitchell JR, Wood E, Collins K (1999) A telomerase component is defective in the human disease dyskeratosis congenita. Nature 402: 551–555. 10591218 10.1038/990141

[9] Vulliamy TJ, Marrone A, Knight SW, Walne A, Mason PJ, et al (2006) Mutations in dyskeratosis congenita: their impact on telomere length and the diversity of clinical presentation. Blood 107: 2680–2685. 16332973 10.1182/blood-2005-07-2622

[10] Yamaguchi H, Calado RT, Ly H, Kajigaya S, Baerlocher GM, et al (2005) Mutations in TERT, the gene for telomerase reverse transcriptase, in aplastic anemia. N Engl J Med 352: 1413–1424. 15814878 10.1056/NEJMoa042980

[11] Armanios MY, Chen JJ, Cogan JD, Alder JK, Ingersoll RG, et al (2007) Telomerase mutations in families with idiopathic pulmonary fibrosis. N Engl J Med 356: 1317–1326. 17392301 10.1056/NEJMoa066157

[12] Alder JK, Chen JJ, Lancaster L, Danoff S, Su SC, et al (2008) Short telomeres are a risk factor for idiopathic pulmonary fibrosis. Proc Natl Acad Sci U S A 105: 13051–13056. 18753630 10.1073/pnas.0804280105 PMC2529100

[13] Diaz de Leon A, Cronkhite JT, Katzenstein AL, Godwin JD, Raghu G, et al. Telomere lengths, pulmonary fibrosis and telomerase (TERT) mutations. PLoS One 5: e10680. 10.1371/journal.pone.0010680 PMC2873288 20502709

[14] Maher TM, Wells AU, Laurent GJ (2007) Idiopathic pulmonary fibrosis: multiple causes and multiple mechanisms? Eur Respir J 30: 835–839. 17978154 10.1183/09031936.00069307

[15] Selman M, King TE, Pardo A (2001) Idiopathic pulmonary fibrosis: prevailing and evolving hypotheses about its pathogenesis and implications for therapy. Ann Intern Med 134: 136–151. 11177318 10.7326/0003-4819-134-2-200101160-00015

[16] Drakopanagiotakis F, Xifteri A, Polychronopoulos V, Bouros D (2008) Apoptosis in lung injury and fibrosis. Eur Respir J 32: 1631–1638. 19043009 10.1183/09031936.00176807

[17] Wang Y, Kuan PJ, Xing C, Cronkhite JT, Torres F, et al (2009) Genetic defects in surfactant protein A2 are associated with pulmonary fibrosis and lung cancer. Am J Hum Genet 84: 52–59. 19100526 10.1016/j.ajhg.2008.11.010 PMC2668050

[18] Marshall RP, Puddicombe A, Cookson WO, Laurent GJ (2000) Adult familial cryptogenic fibrosing alveolitis in the United Kingdom. Thorax 55: 143–146. 10639533 10.1136/thorax.55.2.143 PMC1745672

[19] Mushiroda T, Wattanapokayakit S, Takahashi A, Nukiwa T, Kudoh S, et al (2008) A genome-wide association study identifies an association of a common variant in TERT with susceptibility to idiopathic pulmonary fibrosis. J Med Genet 45: 654–656. 18835860 10.1136/jmg.2008.057356

[20] Tsakiri KD, Cronkhite JT, Kuan PJ, Xing C, Raghu G, et al (2007) Adult-onset pulmonary fibrosis caused by mutations in telomerase. Proc Natl Acad Sci U S A 104: 7552–7557. 17460043 10.1073/pnas.0701009104 PMC1855917

[21] Fauce SR, Jamieson BD, Chin AC, Mitsuyasu RT, Parish ST, et al (2008) Telomerase-based pharmacologic enhancement of antiviral function of human CD8+ T lymphocytes. J Immunol 181: 7400–7406. 18981163 10.4049/jimmunol.181.10.7400 PMC2682219

[22] Liu Y, Snow BE, Hande MP, Yeung D, Erdmann NJ, et al (2000) The telomerase reverse transcriptase is limiting and necessary for telomerase function in vivo. Curr Biol 10: 1459–1462. 11102810 10.1016/s0960-9822(00)00805-8

[23] Dasari A, Bartholomew JN, Volonte D, Galbiati F (2006) Oxidative stress induces premature senescence by stimulating caveolin-1 gene transcription through p38 mitogen-activated protein kinase/Sp1-mediated activation of two GC-rich promoter elements. Cancer Res 66: 10805–10814. 17108117 10.1158/0008-5472.CAN-06-1236 PMC4288740

[24] Volonte D, Galbiati F (2009) Caveolin-1, cellular senescence and pulmonary emphysema. Aging (Albany NY) 1: 831–835. 20157570 10.18632/aging.100079 PMC2815740

[25] Allsopp RC, Cheshier S, Weissman IL (2001) Telomere shortening accompanies increased cell cycle activity during serial transplantation of hematopoietic stem cells. J Exp Med 193: 917–924. 11304552 10.1084/jem.193.8.917 PMC2193408

[26] Fust A, LeBellego F, Iozzo RV, Roughley PJ, Ludwig MS (2005) Alterations in lung mechanics in decorin-deficient mice. Am J Physiol Lung Cell Mol Physiol 288: L159–166. 15447936 10.1152/ajplung.00089.2004

[27] Mehrad B, Burdick MD, Strieter RM (2009) Fibrocyte CXCR4 regulation as a therapeutic target in pulmonary fibrosis. Int J Biochem Cell Biol 41: 1708–1718. 19433312 10.1016/j.biocel.2009.02.020 PMC2681415

[28] Debacq-Chainiaux F, Erusalimsky JD, Campisi J, Toussaint O (2009) Protocols to detect senescence-associated beta-galactosidase (SA-betagal) activity, a biomarker of senescent cells in culture and in vivo. Nat Protoc 4: 1798–1806. 20010931 10.1038/nprot.2009.191

[29] Stellato C, Beck LA, Gorgone GA, Proud D, Schall TJ, et al (1995) Expression of the chemokine RANTES by a human bronchial epithelial cell line. Modulation by cytokines and glucocorticoids. J Immunol 155: 410–418. 7541423

[30] Nichols WW, Murphy DG, Cristofalo VJ, Toji LH, Greene AE, et al (1977) Characterization of a new human diploid cell strain, IMR-90. Science 196: 60–63. 841339 10.1126/science.841339

[31] Roth A, Harley CB, Baerlocher GM (2010) Imetelstat (GRN163L)–telomerase-based cancer therapy. Recent Results Cancer Res 184: 221–234. 20072842 10.1007/978-3-642-01222-8_16

[32] Fridlender ZG, Cohen PY, Golan O, Arish N, Wallach-Dayan S, et al (2007) Telomerase activity in bleomycin-induced epithelial cell apoptosis and lung fibrosis. Eur Respir J 30: 205–213. 17504800 10.1183/09031936.00009407

[33] Park JI, Venteicher AS, Hong JY, Choi J, Jun S, et al (2009) Telomerase modulates Wnt signalling by association with target gene chromatin. Nature 460: 66–72. 19571879 10.1038/nature08137 PMC4349391

[34] Keane MP, Belperio JA, Moore TA, Moore BB, Arenberg DA, et al (1999) Neutralization of the CXC chemokine, macrophage inflammatory protein-2, attenuates bleomycin-induced pulmonary fibrosis. J Immunol 162: 5511–5518. 10228032

[35] Mallick S (2008) Outcome of patients with idiopathic pulmonary fibrosis (IPF) ventilated in intensive care unit. Respir Med 102: 1355–1359. 18635345 10.1016/j.rmed.2008.06.003

[36] Stack BH, Choo-Kang YF, Heard BE (1972) The prognosis of cryptogenic fibrosing alveolitis. Thorax 27: 535–542. 4538877 10.1136/thx.27.5.535 PMC470541

[37] Moeller A, Ask K, Warburton D, Gauldie J, Kolb M (2008) The bleomycin animal model: a useful tool to investigate treatment options for idiopathic pulmonary fibrosis? Int J Biochem Cell Biol 40: 362–382. 17936056 10.1016/j.biocel.2007.08.011 PMC2323681

[38] Kottmann RM, Hogan CM, Phipps RP, Sime PJ (2009) Determinants of initiation and progression of idiopathic pulmonary fibrosis. Respirology 14: 917–933. 19740254 10.1111/j.1440-1843.2009.01624.x PMC3884519

[39] Liu T, Chung MJ, Ullenbruch M, Yu H, Jin H, et al (2007) Telomerase activity is required for bleomycin-induced pulmonary fibrosis in mice. J Clin Invest 117: 3800–3809. 18008008 10.1172/JCI32369 PMC2075478

[40] Lee J, Reddy R, Barsky L, Scholes J, Chen H, et al (2009) Lung alveolar integrity is compromised by telomere shortening in telomerase-null mice. Am J Physiol Lung Cell Mol Physiol 296: L57–70. 18952756 10.1152/ajplung.90411.2008 PMC2636955

[41] Liu T, Hu B, Chung MJ, Ullenbruch M, Jin H, et al (2006) Telomerase regulation of myofibroblast differentiation. Am J Respir Cell Mol Biol 34: 625–633. 16424384 10.1165/rcmb.2005-0252OC PMC2644224

[42] Liu T, Nozaki Y, Phan SH (2002) Regulation of telomerase activity in rat lung fibroblasts. Am J Respir Cell Mol Biol 26: 534–540. 11970904 10.1165/ajrcmb.26.5.4668

[43] Mazzucchelli GD, Gabelica V, Smargiasso N, Fleron M, Ashimwe W, et al (2008) Proteome alteration induced by hTERT transfection of human fibroblast cells. Proteome Sci 6: 12. 18419814 10.1186/1477-5956-6-12 PMC2386453

[44] Shivshankar P, Boyd AR, Le Saux CJ, Yeh IT, Orihuela CJ (2011) Cellular senescence increases expression of bacterial ligands in the lungs and is positively correlated with increased susceptibility to pneumococcal pneumonia. Aging Cell 10: 798–806. 21615674 10.1111/j.1474-9726.2011.00720.x PMC3173515

[45] Verma S, Slutsky AS (2007) Idiopathic pulmonary fibrosis–new insights. N Engl J Med 356: 1370–1372. 17392309 10.1056/NEJMcibr070490

[46] Thannickal VJ, Loyd JE (2008) Idiopathic pulmonary fibrosis: a disorder of lung regeneration? Am J Respir Crit Care Med 178: 663–665. 18796651 10.1164/rccm.200807-1127ED

