# Supplementary figures and images for: A Novel Telomerase Activator Suppresses Lung Damage in a Murine Model of Idiopathic Pulmonary Fibrosis
Source: PLoS One. 2013 Mar 14;8(3):e58423. doi: 10.1371/journal.pone.0058423 (PMC3597721; doi:10.1371/journal.pone.0058423)

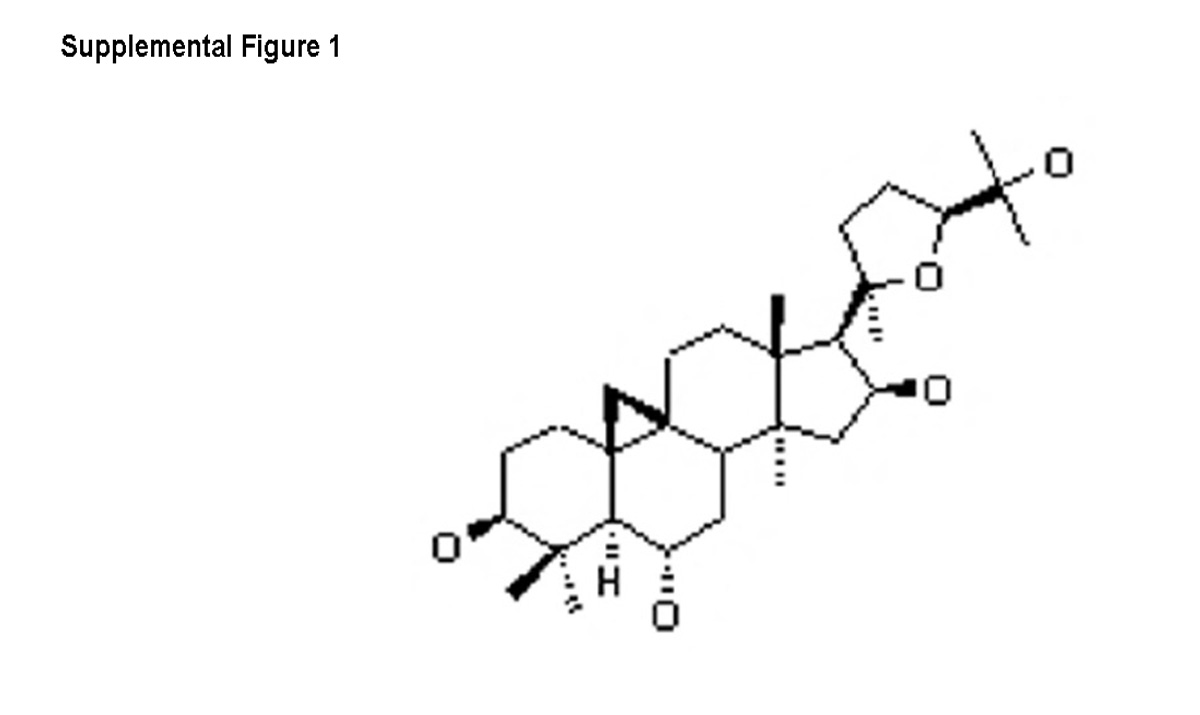

Supplement: Figure S1 — Structure of the small molecule telomerase activator cycloastragenol (GRN510). (TIF) [file pone.0058423.s001.tif]

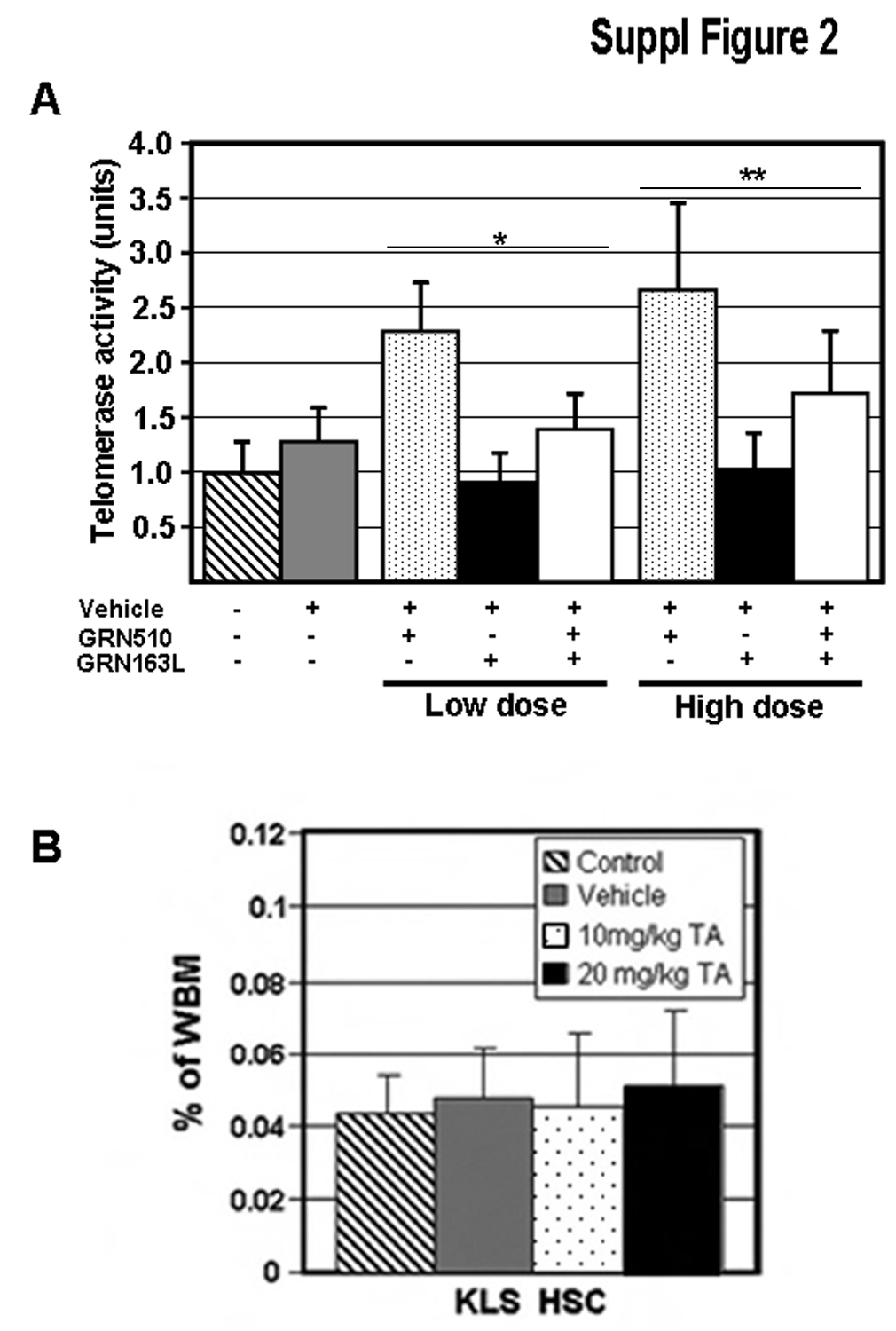

Supplement: Figure S2 — The small molecule GRN510 activates telomerase in vivo. A. Tert Het mice were dosed at either 10 or 20 mg/kg/day for 3 weeks, and bone marrow progenitor cells (cKit+LinNegSca1+ sub-population, or KLS) were subsequently FACS sorted for analysis of telomerase activity using the TRAP assay. The mean level of activity ± SEM, is shown (n = 4–10 for per group). The average activity level of the untreated control group was arbitrarily assigned a value of 1.0. Asterisks indicate a significant difference amongst indicated groups (* & ** 1-way ANOVA ≤0.04; Student's t test P≤0.01 for GRN510 treated groups versus vehicle control). B. FACS analysis of bone marrow progenitor cell numbers after 3 weeks of treatment for the same mice as assessed in A. WBM- whole bone marrow. TA- GRN510. (TIF) [file pone.0058423.s002.tif]

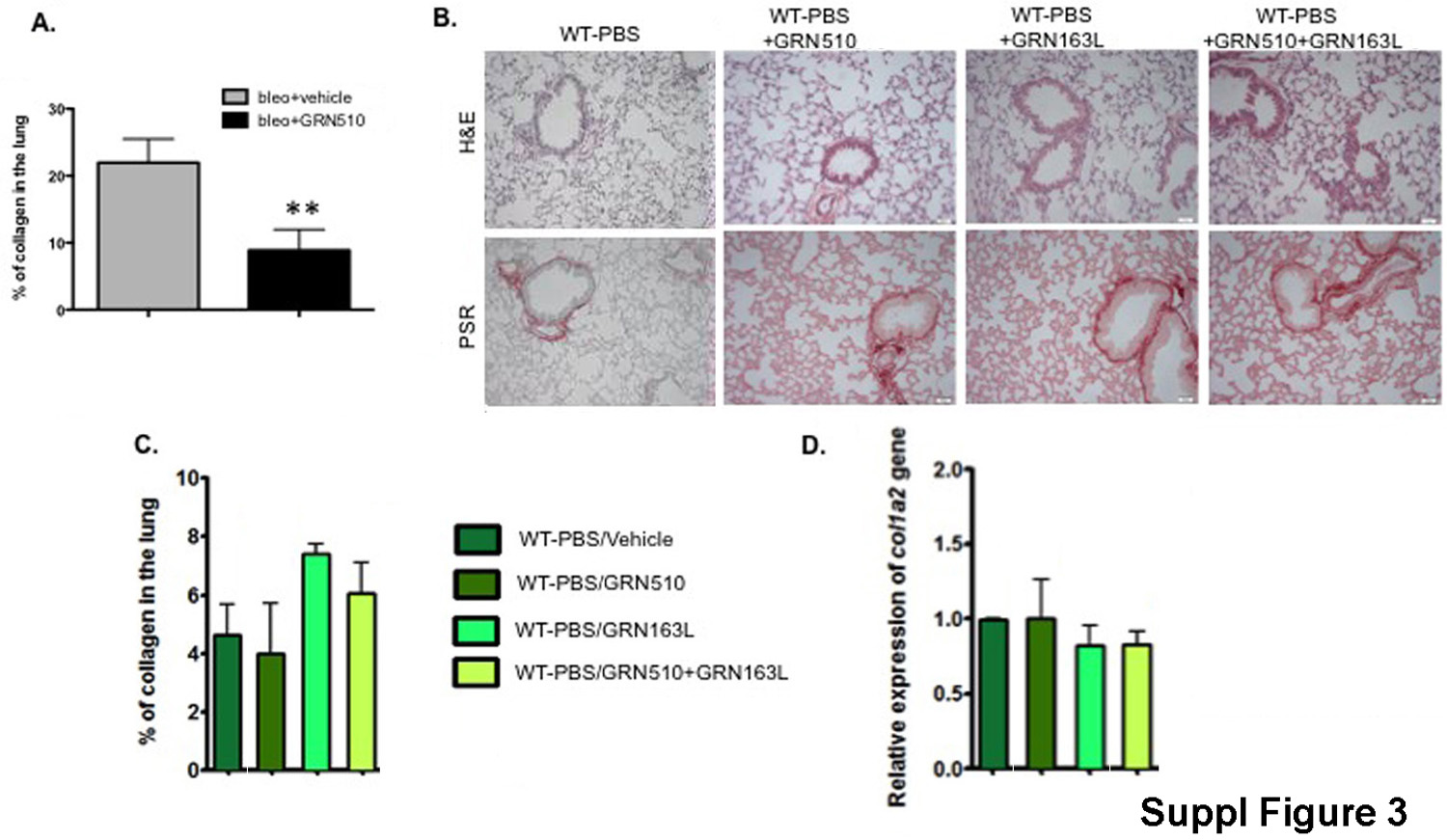

Supplement: Figure S3 — Histologic examination and collagen deposition levels in Wild type PBS control mice. A) Wild type mice were dosed with GRN510 (10 mg/kg/d) for 3 weeks and subjected to bleomycin-induced fibrosis (day 2). Levels of picrosirius red staining were quantified with ImagePro software. * denotes significance between indicated groups (P = 0.007; Student t Test). N = 8–10 animals per group. B) Representative photomicrographs of H&E and PSR-stained lung sections of wild type PBS control mice treated along with GRN510 and/or GRN163L showing no changes in gross pathology and collagen deposition. C) Freshly synthesized collagen levels detected by Sircoll assay in wild type PBS control mice (same mice as in B) show no significant difference. D) Relative mRNA levels of collagen expression in Wild type PBS control mice (same mice as in B). For all plots, N = 4 animals per group. Mean ± SEM is shown. One-way ANOVA revealed no significant difference in collagen levels amongst the groups. (TIF) [file pone.0058423.s003.tif]

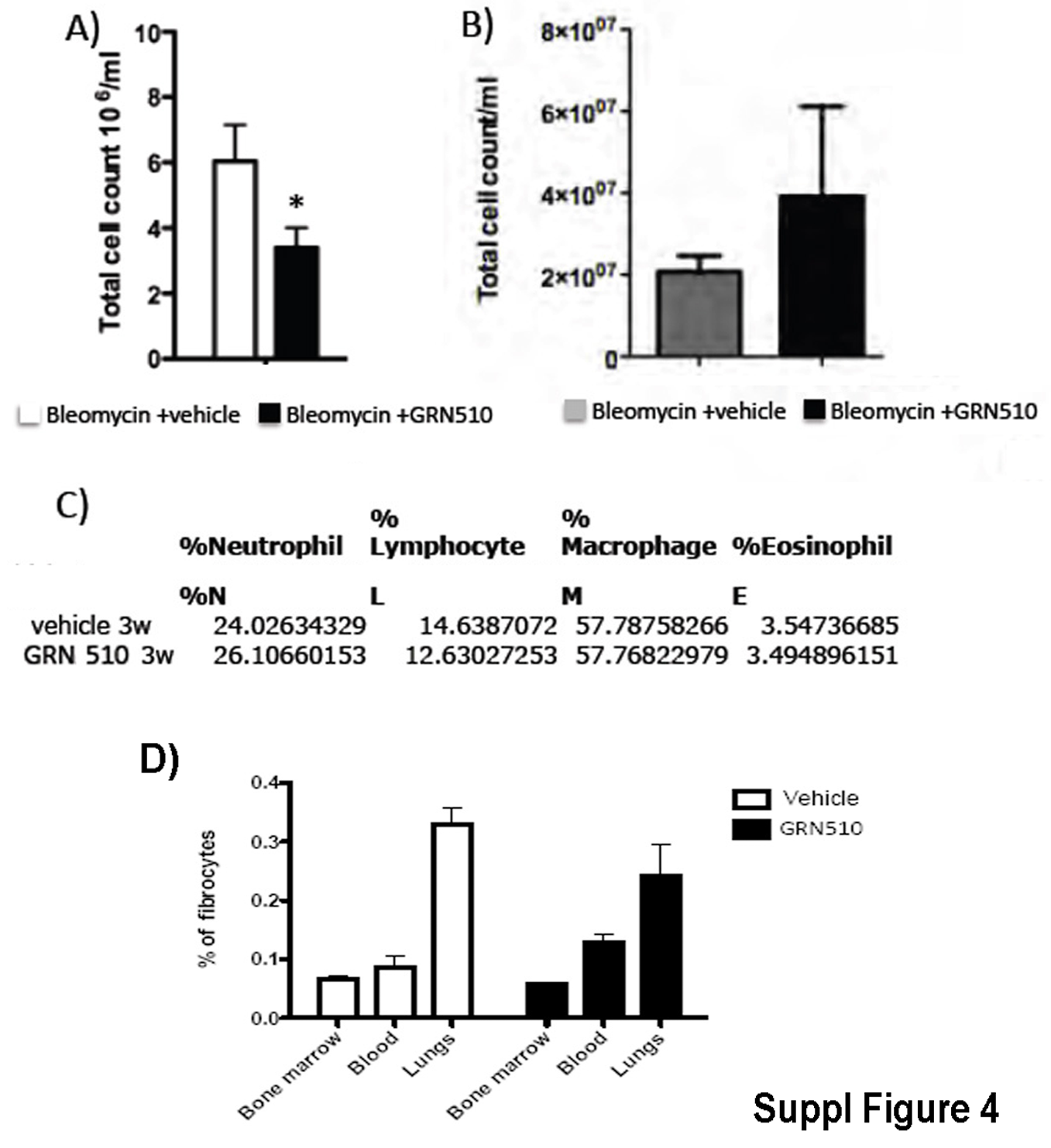

Supplement: Figure S4 — Analysis of the effect of GRN510 on levels of infiltrating leucocytes following Bleomycin induced lung injury. Analysis was performed using Tert Het (A and C; the same mice used in Figure 4, n = 5−10 mice per group) and wild type mice (B; the same mice as used in Suppl Figure S4A, n = 8−10 animals per group). D) Fibrocyte analysis was performed at 11 days post-bleomycin injury in the indicated tissue (the same mice used in figure 4). All GRN510 treated mice received 10 mg/kg/day. Values given are mean ± SEM; asterisk * denotes significance between indicated groups; P = 0.02, Student’s t Test. (TIF) [file pone.0058423.s004.tif]

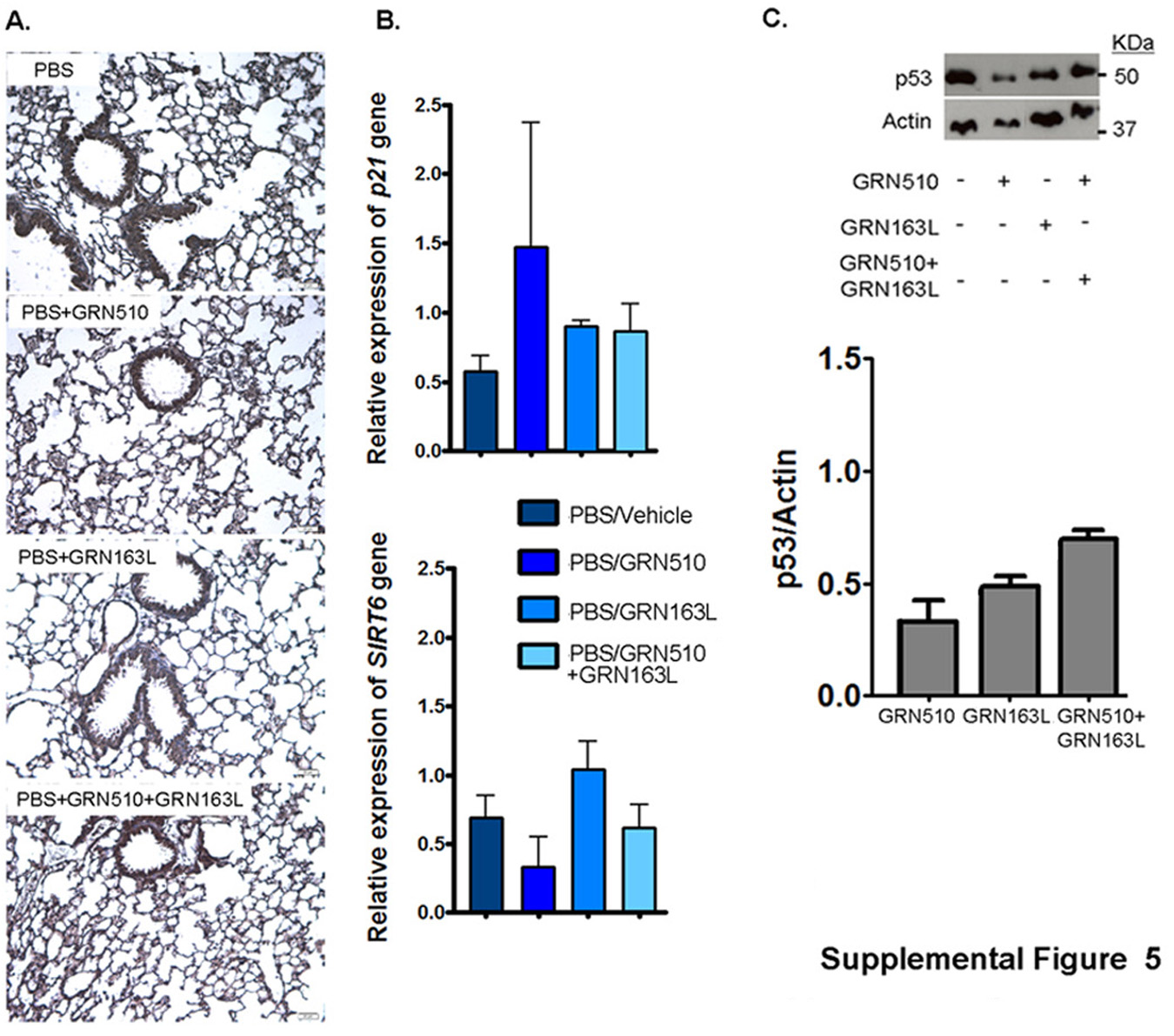

Supplement: Figure S5 — Senescence markers levels in Tert−/+ PBS control mice. A) Representative photomicrographs of lung sections, from Tert−/+ PBS control mice treated with GRN510 (10 mg/kg/d) and/or GRN163L (13 mg/kg/d) for 3 weeks, showing no apparent changes in the MH2A levels. B) Relative mRNA levels of p21 and sirt6 expressions in Tert−/+ -PBS control mice treated along with GRN510 and/or GRN163L. One-way ANOVA revealed no significant difference in collagen levels amongst the groups. C) Western blot analysis and densitometric quantitations of p53 show no significant difference in the levels of p53 in lung homogenates isolated from mice treated with GRN510 and/or GRN163L. For all plots, mean ± SEM is shown; N = 4 animals per group (same mice as analyzed in Figure 2). (TIF) [file pone.0058423.s005.tif]
